# Research on a Cognitive Distraction Recognition Model for Intelligent Driving Systems Based on Real Vehicle Experiments

**DOI:** 10.3390/s20164426

**Published:** 2020-08-07

**Authors:** Qinyu Sun, Chang Wang, Yingshi Guo, Wei Yuan, Rui Fu

**Affiliations:** School of Automobile, Chang’an University, Xi’an 710064, China; sunqinyu@chd.edu.cn (Q.S.); guoys@chd.edu.cn (Y.G.); yuanwei@chd.edu.cn (W.Y.); furui@chd.edu.cn (R.F.)

**Keywords:** intelligent driving system, cognitive distraction driving, wavelet packet analysis, long short-term memory network, attention mechanism

## Abstract

The accurate and prompt recognition of a driver’s cognitive distraction state is of great significance to intelligent driving systems (IDSs) and human-autonomous collaboration systems (HACSs). Once the driver’s distraction status has been accurately identified, the IDS or HACS can actively intervene or take control of the vehicle, thereby avoiding the safety hazards caused by distracted driving. However, few studies have considered the time–frequency characteristics of the driving behavior and vehicle status during distracted driving for the establishment of a recognition model. This study seeks to exploit a recognition model of cognitive distraction driving according to the time–frequency analysis of the characteristic parameters. Therefore, an on-road experiment was implemented to measure the relative parameters under both normal and distracted driving via a test vehicle equipped with multiple sensors. Wavelet packet analysis was used to extract the time–frequency characteristics, and 21 pivotal features were determined as the input of the training model. Finally, a bidirectional long short-term memory network (Bi-LSTM) combined with an attention mechanism (Atten-BiLSTM) was proposed and trained. The results indicate that, compared with the support vector machine (SVM) model and the long short-term memory network (LSTM) model, the proposed model achieved the highest recognition accuracy (90.64%) for cognitive distraction under the time window setting of 5 s. The determination of time–frequency characteristic parameters and the more accurate recognition of cognitive distraction driving achieved in this work provide a foundation for human-centered intelligent vehicles.

## 1. Introduction

Distracted driving has developed as one of the dominating inducements of crashes [1], and happens when a driver consciously or unconsciously transfers their attention from the main driving operation to other tasks unrelated to driving; this attention shift impairs the driver’s scenario perception, decision-making, and manipulative effects [2]. With the widespread use of information media such as in-vehicle information systems and cell phones, more and more distracted driving has appeared and seriously threatens traffic safety [3]. It is evident that distraction severely impacts driving safety. Therefore, for intelligent driving systems (IDSs), determining how to effectively detect and recognize driver distraction is the key to, and prerequisite for, taking intervention measures [4]. 

Distracted driving is usually categorized as one of three types, namely operational distraction, visual distraction, and cognitive distraction [5]. Operational distraction refers to the transfer of the driver’s senses or locomotive organs from the vehicle handling structure required by the main driving task to other places, such as by shifting hands from the steering wheel, resulting in the driver being unable to safely manipulate the vehicle [6]. Visual distraction means that the driver’s sight intentionally or unintentionally leaves the road and shifts to something unrelated to driving [7]. Cognitive distraction refers to the driver reflecting on other tasks unrelated to driving, which makes the driver unable to drive safely or impairs his or her reaction ability [8]. However, both visual and operation distraction will inevitably be accompanied by cognitive distraction, the impact of which on driving safety is more complex and subtle. In their summary of the impacts of disparate classifications of distraction on driving safety, Hagiwara et al. [9] pointed out that assessing the influence of cognitive distraction on driving safety must be the focus of future research. In addition, compared to cognitive distraction, the visual distraction and operational distraction can be more easily recognized by IDSs; moreover, drivers themselves are more likely to be aware of the risks of visual and operational distraction, but lack of awareness of the extent of the danger of cognitive distraction or easily ignore the impact of cognitive distraction on driving safety [10]. Most related studies have not conducted in-depth research on the recognition of cognitive distraction.

The core of the construction of a recognition model of cognitive distraction is to extract the features of the vehicle’s state and driver operation data during the driver’s cognitive distraction to provide a basis for model establishment. Ranney et al. [11] required test subjects to complete simple tasks, difficult tasks, and basic tasks as quickly as possible while maintaining a constant car-following distance, and the results revealed that the steering wheel rotation rate increased when performing secondary tasks, thereby indicating cognitive distraction. During a study of the driving behaviors of drivers while they made mathematical calculations, Shi et al. [12] proposed that the number of steering wheel turns and the number of pedal depressions increased with the increased level of brain load. Overall, cognitive distraction may lead to poorer steering stability. Harbluk et al. [13] used the number of emergency braking events to represent the driver’s longitudinal control ability, and found that the number of emergency braking events increased significantly with the promotion of the difficulty of cognitive distraction tasks. Peng et al. [14] studied the influence of cognitive distraction caused by an on-board information system on vehicle longitudinal control ability via a simulator. The results indicated that the cognitive distraction caused by both text input and text reading tasks led to significant increases (45% and 30%, respectively) in the standard deviation of the headway time as compared with normal driving. The standard deviation of lane position (SDLP) is generally employed to describe the lateral control ability. Liang et al. [15] manifested that the SDLP decreased and the lane-keeping characteristics improved when drivers performed cognitive distraction tasks. Jan et al. [16] proposed that the driver’s gaze point would be more concentrated and the lane-keeping ability would be improved via driving simulator and real vehicle experiments. Through an on-road test of skilled drivers, Deram et al. [17] confirmed that the steering wheel angle, lateral position, lateral speed, and steering wheel angular speed of the vehicle during cognitive distraction driving were significantly different from those during normal driving, and can therefore be employed to characterize cognitive distraction. 

Cognitive distraction recognition has been the theme of abundant empirical research that has analyzed diverse characterization parameters and machine learning algorithms to establish identification models. Fagerberg et al. [18] implemented on-road tests and detected the distracted state of drivers via the vehicle speed, steering wheel angular speed, and steering signal. Yang et al. [19] used a global positioning system (GPS) to collect data on the vehicle speed, yaw angle, lateral position, and longitudinal position, then employed Gaussian mixture models (GMMs) to identify cognitive distraction behavior; the average recognition rate reached 70%. Yekhshatyan [20] detected distraction via the driver’s visual behavior and vehicle operating state, and it was revealed that the combination of these factors can promote the competence of machine learning models to identify visual and cognitive distraction. Kutila et al. [21] combined eye movement and lane-keeping characteristics to detect the cognitive distraction state of drivers, and used a support vector machine (SVM) to classify the collected data; it was found to be able to detect 80% of visual distraction and 68%–86% of cognitive distraction. He et al. [22] used multiwavelet transform and Fourier transform to analyze data on the steering wheel angle collected from a driving simulator test, and proved that it is effective and feasible to employ the wavelet transform of the steering wheel angle data to detect the driver’s mental state by employing the chaos theory analysis method. Zhao et al. [23] extracted the energy characteristics of dangerous driving behavior parameters in different frequency bands via multiwavelet analysis, and combined the time domain, spatial information, and phase to construct the characteristic parameters of dangerous driving behavior. Finally, an SVM was employed to classify the driving status. The digital memory and logical reasoning served as secondary tasks in the study from Jin et al. [24] for the analysis of normal driving and cognitive distraction driving characteristics via a driving simulator. The vehicle speed, vehicle acceleration, vehicle yaw velocity, steering wheel angle, and steering wheel angular velocity were collected as characteristic parameters to recognize cognition distraction, and the results revealed that the recognition rate of the straight line reached 88.58%.

At present, the overwhelming majority of cognitive distraction recognition models are established based on the analysis of time domain features [25,26,27]. To explore whether a driver is in a cognitive distraction status via the operational behavior and vehicle movement data, the key is to determine characteristic parameters that can distinguish normal driving from distracted driving. However, the driving behavior and vehicle movement signals are not stable, which manifests that the mean, variance, and covariance of the collected related parameters will transform over time. It is often imperative to know the trend of the variation of the signal spectrum with time when processing non-stationary time series data, i.e., the time–frequency characteristic of the signal [28,29]. Therefore, it is not sufficient to homogeneously construct a recognition model by extracting the cognitive distraction characteristic parameters from the time domain or frequency domain.

To address the deficiencies in the recognition models of cognitive distraction for IDSs, in the present research, the six-layer wavelet packet of the collected driving behavior and vehicle state parameters were decomposed and reconstructed from the perspective of time–frequency characteristic analysis through the implementation of the wavelet package analysis method. The distribution characteristics of the relative energy of the corresponding frequency bands during normal driving and cognitive distraction driving were studied, and the frequency band energy with a significant difference was determined as the characteristic index. On this basis, a bi-directional long short-term memory (Bi-LSTM) network combined with an attention model (Atten-BiLSTM) was employed to establish a recognition model of cognitive distraction. In addition, on-road experiments of a real vehicle were designed and implemented to obtain cognitive distraction driving data under the designed cognitive distraction tasks. Calculation questions and the memorization of phone numbers were selected as distraction subtasks. The driving behavior and vehicle state parameters included the steering wheel angle, steering wheel angular velocity, vehicle speed, vehicle yaw rate, and vehicle longitudinal and lateral acceleration, and data on these parameters while the drivers were engaged in different subtasks were collected. Finally, the feature sequences of cognitive distraction from on-road tests were employed to train the identification model and perform offline experimental verification.

**Figure 1:**
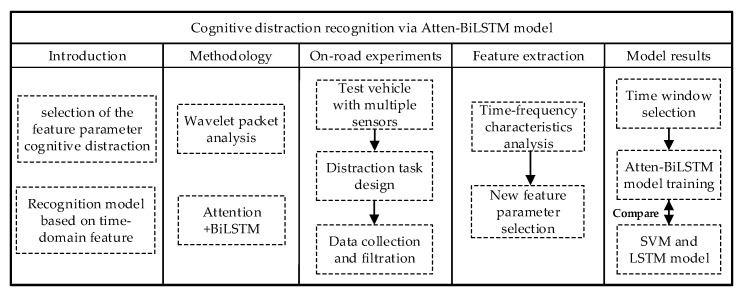
Cognitive distraction recognition framework.

The remainder of the paper is structured as follows. Section 2 details the methods used in this work, including wavelet packet analysis and the Atten-BiLSTM network. Section 3 provides detailed information on the experimental design, process, equipment, and sensors. Section 4 exhibits the results of the time–frequency analysis of the data of characteristic parameters collected when drivers were cognitively distracted. The recognition results of cognitive distraction from the Atten-BiLSTM, SVM, and LSTM methods are introduced in Section 5. Finally, discussions and conclusions are exhibited in Section 6. The main framework of this study is presented in Figure 1.

## 2. Method

Time–frequency characteristic analysis is pivotal to obtain non-stationary time series features. However, scarce research has focused on the time–frequency characteristics of the driving behavior and vehicle status during distracted driving for the establishment of a recognition model. Therefore, in this study, wavelet packet analysis was employed to extract the time–frequency characteristics of the collected data including the steering wheel angle, steering wheel angular velocity, vehicle speed, vehicle yaw rate, and vehicle longitudinal and lateral acceleration. By using the six-layer decomposition algorithm, new features based on the time–frequency analysis could be extracted from the time series data, which can provide support for improving the accuracy of the recognition model. In addition, traditional machine learning algorithms have been widely used in the establishment of distraction recognition models. This article employed the deep learning algorithms to train the time–frequency features of cognitive distractions and establish a recognition model. Among the deep learning algorithms, Bi-LSTM has great advantages in processing long-term sequences. Therefore, this paper determined the Bi-LSTM algorithm as the foundation for the establishment of the recognition model. In order to further improve the performance of the recognition model, this paper brought in the attention mechanism based on the Bi-LSTM model, thereby increasing the model’s attention to the pivotal features, and then promoting the recognition accuracy of the cognitive distraction recognition model. 

### 2.1. Wavelet Packet Analysis

Wavelet analysis theory is a new function approximation tool and a novel approach of time–frequency analysis and is the consequence of the inheritance and development of Fourier analysis theory [30]. Fourier transform can only separately analyze data from the time domain or the frequency domain, and cannot combine the two domains to observe the signal. Wavelet transform analyzes the signal via a window function called the wavelet function, which is the same as the short-time Fourier transform [31]. However, a significant peculiarity of the wavelet transform is that the local characteristics of the signal could be analyzed together with both the time and frequency domains. Another peculiarity is multi-resolution analysis, i.e., the resolutions of frequency and time can be changed according to varied requirements. In wavelet transform, at high signal frequencies, the frequency resolution decreases and the time resolution increases; on the contrary, the frequency resolution increases and the time resolution decreases at low signal frequencies [32]. Therefore, wavelet transform was employed in the present study to extract the time–frequency characteristics of driving behavior and vehicle status data when drivers were cognitively distracted, and new characteristic parameters were determined. Therefore, this study ameliorates the research method of cognitive distraction and provides a basis for the establishment of a recognition model.

According to the research results of multi-resolution analysis, if the standard orthogonal basis in $S^{2}\left(O\right)$ is composed of binary discrete wavelet function clusters $\{\xi_{j,n}\left(t\right);j,n\in Z\}$, the orthogonal wavelet decomposition of the input data $x\left(t\right)\in S^{2}\left(O\right)$ is as follows:

(1) $$x\left(t\right)=\overset{M}{\underset{j=1}{\sum }}\underset{n\in Z}{\sum }a_{n}^{j}\xi_{j,n}\left(t\right)+\underset{n\in Z}{\sum }b_{n}^{M}\vartheta_{j,n}\left(t\right),$$

where M is the number of decompositions, $d_{n}^{j}$ is the coefficient of wavelet decomposition, $b_{n}^{M}$ is the coefficient of scale decomposition, $\xi_{j,n}\left(t\right)$ and $\vartheta_{j,n}\left(t\right)$ are a cluster if binary orthogonal functions are determined by the basic wavelet function ξ(t) and a basic scale function ϑ(t), respectively; and $a_{n}^{j}$ and $b_{n}^{M}$ meet the following recursive decomposition criterion.

(2) $$\{\begin{array}{c} a_{n}^{j+1}=\underset{l\in Z}{\sum }b_{l}^{j}h_{1\left(l-2n\right)}\\ b_{n}^{j+1}=\underset{l\in Z}{\sum }b_{l}^{j}h_{0\left(l-2n\right)} \end{array}$$

where $h_{0n}$ and $h_{1n}$ are two filters that satisfy the two-scale difference equation, as follows.

(3) $$\{\begin{array}{c} \xi (t)=\sqrt{2}\underset{n\in Z}{\sum }h_{0n}\xi \left(2t-n\right)\\ \vartheta (t)=\sqrt{2}\underset{n\in Z}{\sum }h_{1n}\vartheta \left(2t-n\right) \end{array}$$

Wavelet packet analysis can decompose the frequency band at multiple levels. To enhance the time–frequency resolution, the high-frequency part is further decomposed with a lower-frequency resolution during wavelet analysis. Let ξ(t) and ϑ(t) satisfy the two-scale criterions. Note that $\lambda_{0}(t)=\xi (t)$, $\lambda_{1}(t)=\vartheta (t)$, and the definitions are as follows.

(4) $$\{\begin{array}{c} \lambda_{2m}\left(t\right)=\sqrt{2}\underset{n\in Z}{\sum }h_{0n}\lambda_{m}\left(2t-n\right)\\ \lambda_{2m+1}\left(t\right)=\sqrt{2}\underset{n\in Z}{\sum }h_{1n}\lambda_{m}\left(2t-n\right) \end{array}$$

The orthogonal wavelet packet is the functional system $\{\lambda_{m}{\left(t\right)\}}_{m\in Z}$, which is determined by $\lambda_{0}(t)=\xi (t)$. Therefore, the wavelet packet $\lambda_{0}(t)=\xi (t)$ is a set of related functions including the scale function $\lambda_{0}(t)$ and the wavelet function $\lambda_{1}(t)$. By applying the orthogonal wavelet decomposition algorithm to the wavelet packet, the seasoning relationship of the wavelet packet decomposition algorithm is as follows.

(5) $$a_{n}^{j+1,2m}=\underset{l\in Z}{\sum }a_{l}^{j,m}h_{0\left(l-2n\right)}$$

(6) $$a_{n}^{j+1,2m+1}=\underset{l\in Z}{\sum }a_{l}^{j,m}h_{1\left(l-2n\right)}$$

Then, the wavelet packet reconstruction algorithm is as follows.

(7) $$a_{n}^{j,m}=\underset{l\in Z}{\sum }a_{l}^{j+1,2m+1}h_{0\left(n-2l\right)}+\underset{l\in Z}{\sum }a_{l}^{j+1,2m}h_{1\left(n-2l\right)}$$

The steps of the feature extraction of frequency band energy after wavelet packet decomposition mainly include the following (three-layer decomposition is used as an example for illustration). First, the wavelet basis function is selected to perform three-layer decomposition on the original signal. Then, the signal is reconstructed according to the decomposition coefficient obtained in the previous step, and the formula for expressing the original signal with the reconstructed signal is as follows:

(8) $$D=D_{30}+D_{31}+D_{32}+D_{33}+D_{34}+D_{35}+D_{36}+D_{37,}$$

where D is the primitive signal, $D_{ij}$ denotes the reconstructed signal of $X_{ij}$, $X_{ij}$ is the coefficient of wavelet packet decomposition, and (***i,j***) denotes the ***j***-th node in the ***i***-th layer. 

Third, the energy $E_{j}$ of the reestablished data in each frequency band is solved. This is defined as $E_{j}=\overset{m}{\underset{n=1}{\sum }}{\left|D_{3j}\left(n\right)\right|}^{2}$, where the discrete signal amplitude of the reconstructed signal $D_{3j}$ is $D_{3j}\left(n\right),\;n=1,2,\cdots ,m$, and ***m*** represents the extent of the reconstructed data. Finally, $E_{j}$ is normalized; $E_{j}$ represents the energy of the reestablished data, and the total energy is $E=\overset{7}{\underset{j=0}{\sum }}E_{j}^{2}$. The normalized relative energy is as follows.

(9) $$R_{j}=\frac{E_{j}}{E};\;\;\;\;\;\;\;j=0,1\ldots 7$$

The energy gap between different frequency bands is very large. To facilitate observation and comparison, the logarithmic value of normalized energy is taken as the analysis object, as given by the following.

(10) $$En_{j}=\log R_{j};\;\;\;j=0,1\;\ldots 7$$

The wavelet basis function can be divided into orthogonal and non-orthogonal functions. The common orthogonal wavelet foundation functions mainly contain the Harr wavelet, Daubechies wavelet, Coiflets wavelet, and Symlets wavelet, while the non-orthogonal wavelet basis functions mainly involve the Morlet wavelet and Mexican hat wavelet. Orthogonal wavelet basis functions are used in wavelet packet transformation and dyadic wavelet transformation. Both orthogonal and non-orthogonal wavelet foundation functions could be applied in continuous wavelet transformation [33]. In this work, the wavelet packet transform method was employed to deal with the data by employing the Haar wavelet, Daubechies wavelet, and Symlets wavelet, respectively. By comparison, it was found that the influence of the wavelet basis function was less than that of the decomposition layer. Finally, db3 was intended as the wavelet foundation function.

To determine the number of decomposition layers, the relative frequency band energy results of five, six, and seven decompositions and reconstructions of the yaw angular velocity using the db3 wavelet were comparatively analyzed. The results indicate that 32 frequency bands were obtained by the decomposition and reconstruction of the five-layer wavelet packet, each of which had a bandwidth of 0.31 Hz, resulting in fewer frequency bands and a larger bandwidth. There were very few frequency bands with significant differences between different driving states. Additionally, 128 frequency bands were obtained by the decomposition and reconstruction of the seven-layer wavelet packet, each of which had a bandwidth of 0.078 Hz. There were more frequency bands and the bandwidth was also suitable. However, the sampling time required for the seven-layer decomposition was nearly one minute. Under normal circumstances, the time for cognitive distraction to occur is relatively short, and the results obtained by the seven-layer decomposition cannot correspond to the actual situation. Therefore, the six-layer decomposition was ultimately selected for consideration.

### 2.2. Bidirectional Long Short-Term Memory Network

The recurrent neural network (RNN) was established by Seppo for the processing of sequence data, and a parameter-sharing method was employed to enhance the generalization competence of the training network [34]. Although the RNN algorithm has achieved excellent results in various fields, the problems of gradient explosion or disappearance in the backpropagation process have not been effectively improved. To conquer the defects of the RNN algorithm, Hochreiter and Schmidhuber constructed the long short-term memory network (LSTM) according to the RNN structure, and a gate-controlled cell including an input gate, forget gate, and output gate was introduced into the unit [35]. The main function of the gate structure is to selectively delete or add relevant information to the state of the cell to keep it continuously updated. Therefore, the structure effectively addresses the imperfection of the long sequence dependence present in the RNN and enables the networks to have a longer memory ability, thereby ameliorating the gradient explosion and disappearance problems [36]. The specific working procedures of the LSTM model are presented as follows.

(1) Apply the forget gate to delete irrelevant information in the cell unit. The specific information that needs to be deleted is determined by the sigmoid layer in the forget gate. The input of the forget gate is composed of the input data $x_{t}$ of the layer at the current moment and the hidden layer output $h_{t-1}$ at the last moment.

(11) $$f_{t}=\sigma \left(V_{h}\cdot \left[h_{t-1},x_{t}\right]+d_{f}\right)$$

where σ is the sigmoid function of the forget gate, $V_{h}$ is the weight matrix, $d_{f}$ is the bias term, and the output range of $f_{t}$ is [0,1]. The larger the output value, the lesser the degree of forgetting, i.e., the more cell information is retained at the last time. At this moment, the output of the cell will be greatly affected by the cell at the last moment.

(2) Use the input gate to add fresh information to the unit. The specific information that needs to be added is determined by the sigmoid layer and the tanh layer in the input gate, as shown in Equations (12) and (13). The input of the input gate is determined by data $x_{t}$ at the current moment and the hidden layer output $h_{t-1}$ at the last moment.

(12) $$i_{t}=\sigma \left(V_{t}\cdot \left[h_{t-1},x_{i}\right]+d_{i}\right),$$

(13) $${\tilde{C}}_{t}=\tanh\left(V_{c}\cdot \left[h_{t-1},x_{i}\right]+d_{c}\right),$$

where σ is the sigmoid function of the input gate, ***tanh*** is the tanh function, $V_{t}$ and $V_{c}$ are the weight matrixes, $d_{i}$ and $d_{c}$ are the bias terms, $i_{t}$ is the update value of the input gate cell, and ${\tilde{C}}_{t}$ is the update value of the tanh function.

(3) The update value of the cell state can be obtained by combining Equations (11)–(13). As shown in Equation (14), the state value of the original cell is multiplied by the input of the forget gate to delete irrelevant information. The results of the output values of the sigmoid layer and the tanh layer are then combined with the output value of the forget gate to obtain the update value of the unit status $C_{t}$ at the current time.

(14) $$C_{t}=f_{t}\cdot C_{t-1}+i_{t}\cdot {\tilde{C}}_{t},$$

where $C_{t-1}$ is the unit status value at the last moment.

(4) Apply the output gate to transfer the relevant message to the cell at the next moment, which is determined by the sigmoid layer in the output gate and the update value of the cell state. The output of the sigmoid layer in the output gate is shown as follows:

(15) $$o_{t}=\sigma \left(V\cdot \left[h_{t-1},x_{t}\right]+d_{o}\right)$$

where σ is the sigmoid function of the output gate, $V_{o}$ is the weight matrix, and $d_{o}$ is the bias term.

(5) The final output of the unit at the current moment $h_{t}$ can then be expressed as follows.

(16) $$h_{t}=o_{t}\cdot \tan h\left(C_{t}\right)$$

The cell unit in the LSTM network can usually only process information in one direction, while Bi-LSTM can simultaneously process information in both the positive and negative directions, allowing it to obtain more complete information sequence data. Let the input of the Bi-LSTM model at time ***t*** be $x_{t}$. During information processing, the state update of the network layer of the Bi-LSTM model from front to back is as follows:

(17) $${\vec{h}}_{t}=H\left(V_{x{\vec{h}}_{t}}x_{t}+V_{\vec{h}\vec{h}}{\vec{h}}_{t-1}+d_{\vec{h}}\right)$$

where H is the output function of the backward layer, $V_{x{\vec{h}}_{t}}$ is the weight matrix from the input layer to the forward layer, $V_{\vec{h}\vec{h}}$ is the weight matrix between the forward layers, and $d_{\vec{h}}$ is the bias term.

The state update of the network layer from front to back is shown as following.

(18) $${\overset{↼}{h}}_{t}=H^{\prime }\left(V_{x{\overset{↼}{h}}_{t}}x_{t}+V_{\overset{↼}{h}\overset{↼}{h}}{\overset{↼}{h}}_{t-1}+d_{\overset{↼}{h}}\right)$$

where $H^{\prime }$ is the output function of the forward layer, $V_{x{\overset{↼}{h}}_{t}}$ is the weight matrix from the input layer to the backward layer, $V_{\overset{↼}{h}\overset{↼}{h}}$ is the weight matrix between the backward layers, and $d_{\overset{↼}{h}}$ is the bias term.

Then, the output of the Bi-LSTM model after network layer superposition is

(19) $$h_{t}=\tilde{H}\left(V_{\vec{h}o}{\vec{h}}_{t}+V_{\overset{↼}{h}o}{\overset{↼}{h}}_{t}+d_{o}\right)$$

where $\tilde{H}$ is the output function of the forward layer, $V_{\vec{h}o}$ is the weight matrix from the input layer to the backward layer, $V_{\overset{↼}{h}o}$ is the weight matrix between the backward layers, and $d_{o}$ is the bias term.

### 2.3. Bi-LSTM with Attention Mechanism

**Figure 2:**
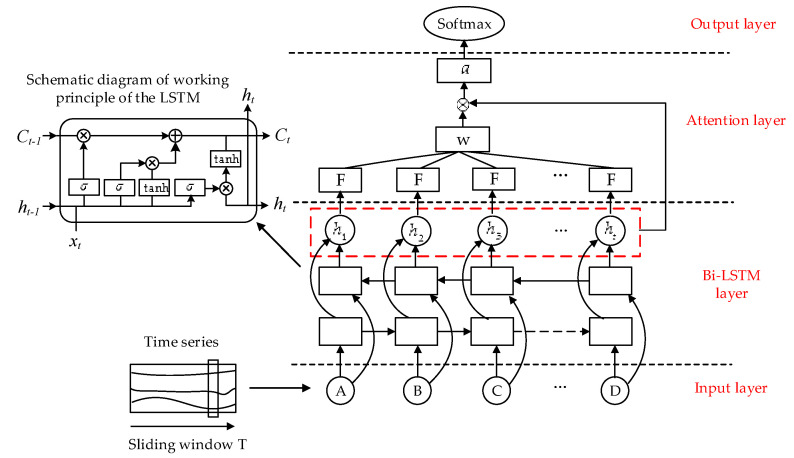
Bidirectional long short-term memory network (Bi-LSTM) model with an attention mechanism.

The attention model is derived from the simulation of the visual signal processing mechanism of the human brain [37]. When the brain is processing visual signals, it will focus on certain areas in the image and extract relevant detailed features from these regions of focus. Similar to this mechanism, the attention mechanism can filter out the parts that have an important impact on the task target from the input data. The key feature information that is screened out can not only reduce the influence of noise on the model training, but also effectively improves the operational efficiency and accuracy of the algorithm [38]. In this work, the problem of a driver’s cognitive distraction recognition is regarded as a modeling and classification problem based on time characteristic sequences. Since the application of the attention mechanism model can ensure that greater weight is distributed to the pivotal characteristics during the modeling process, this can effectively improve the model recognition accuracy. Therefore, the attention model and Bi-LSTM model were combined in this study to establish a recognition model of the cognitive distraction driving of drivers, and the model structure diagram is exhibited in Figure 2. 

As shown in Figure 2, the established model includes four layers, namely the input layer, the Bi-LSTM layer, the attention layer, and the output layer. The input layer includes the features of time series after wavelet packet analysis, and the detailed description of the features selection is exhibited in Section 4. The Bi-LSTM layer is mainly composed of the LSTM models. The schematic diagram of the working principle of the LSTM model is presented in the left of the figure, and the specific calculations of the LSTM model are described in Section 2.2. The Bi-LSTM layer implements preliminary feature extraction on the input data. The attention layer performs linear weighting on the input data (the output of the Bi-LSTM layer) to complete the screening of the pivotal features. When several feature sequences are input, the attention algorithm obtains the weight value of each feature sequence through a similarity calculation. The weight value is employed to denote the attention degree of the attention mechanism to the feature sequences. The larger the weight is, the more attention the algorithm pays to the feature sequence, that is, the greater the influence of the feature after weighted combination. Therefore, in this work, a fully-connected layer is added on the basis of the Bi-LSTM model to realize the learning function. The added learning function ***F*** is employed to calculate the weight coefficient $c_{t}$ of the Bi-LSTM output vector $h_{t}$, and the pivotal feature vector *a* can be calculated by linear weighting. Finally, the softmax function in the output layer is used to output the recognition results. The output of the learning function ***F*** can be expressed as follows:

(20) $$f_{t}=F\left(h_{t}\right).$$

The weight coefficient $c_{t}$ is

(21) $$c_{t}=\frac{exp\left(f_{t}\right)}{\sum_{i=1}^{n}exp\left(f_{t}\right)}.$$

Then, the pivotal feature vector ***a*** can be computed as

(22) $$\overset{n}{\underset{i=1}{\sum }}c_{t}h_{t}.$$

In this study, because cognitive distraction recognition is a two-category problem (normal driving and cognitive distraction driving), the softmax function was selected as the activation function, the Adam algorithm was chosen as the optimizer, and binary_crossentropy was selected as the loss function, and the computational formula is

(23) $$loss=-\overset{n}{\underset{i=1}{\sum }}{\overset{ˇ}{y}}_{i}logy_{i}$$

where ${\overset{ˇ}{y}}_{i}$ denotes the true probability and $y_{i}$ denotes the predicted probability.

During the model training, the total sample set was distributed into a training set, a verification set, and a test set according to the ratio of 6:3:1. The selected feature vectors are described in detail in Section 4. The time window was selected as 5 s, the dropout rate was 0.4, and each layer of the model contained 128 hidden units. The maximum number of epochs was 80. The learning rate in the Adam algorithm was 0.01, and the attenuation value was 0.9. 

## 3. On-Road Experiments

### 3.1. Apparatus

**Figure 3:**
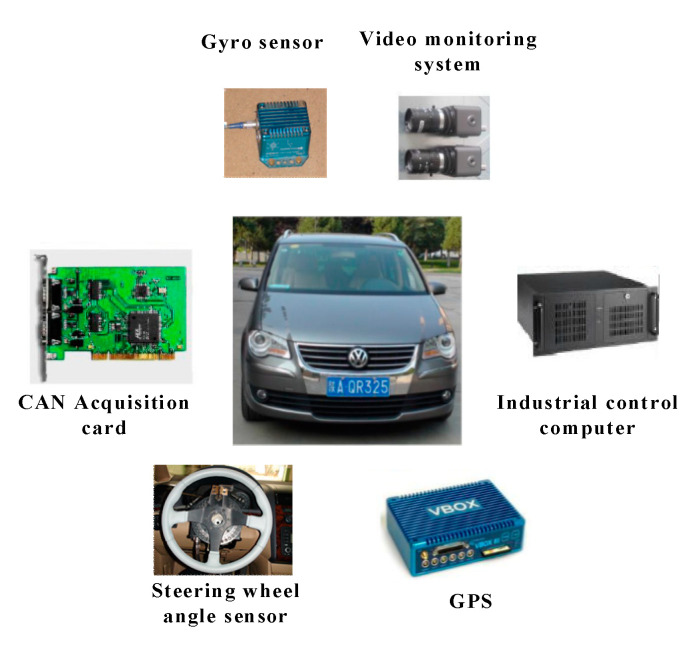
The experimental equipment.

The experimental vehicle (exhibited in Figure 3) applied in the on-road tests was a multi-purpose vehicle, which was equipped with a steering wheel angle sensor for the measurement of the steering wheel angle and angular velocity during normal driving and cognitive distraction driving, and a gyro sensor (IMU02) for the collection of the vehicle status data including the yaw rate, longitudinal acceleration, and lateral acceleration. The vehicle was also equipped with a VBOX (a device that can determine a vehicle’s GPS coordinates) to record the vehicle speed, and a video monitoring system to collect the operation data of the drivers and driving environment. The data collected from the steering wheel angle sensor, gyro sensor, and VBOX were all transmitted through a CAN bus data communication system. 

### 3.2. Participants and Driving Route

Thirty-two drivers (29 males and 3 females) were recruited in the cognitive distraction driving tests. The age range of the drivers was 24 to 51 years old, and the mean age was 36.5 years, with a standard deviation value of 7.62. The driving experience range of the participants was 6 to 29 years, and the average value was 15.4 years, with a standard deviation value of 6.2. The total participants possessed a driver’ license and they were not professional drivers. In addition, all the participants had not undergone a serious crash in the past five years. 

**Figure 4:**
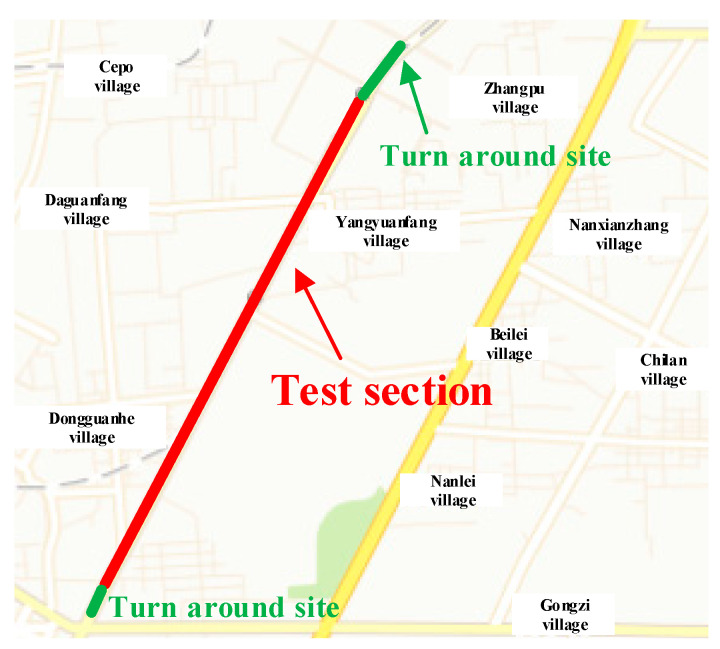
The map of the experimental route.

The choice of the test route has a direct impact on the implementation of a test, as well as the driver’s physiology and psychology, thereby affecting the final test results. After conducting field investigations on multiple road sections and comparing the alignment, traffic conditions, environment, and other factors of each road section, Xitai Road in Xi’an, China was determined as the driving route for the following reasons. First, the selected road is not affected by additional factors such as pedestrian interference, slope, or curvature factors that can have an impact on the drivers. Second, the test route is not too long, and the road environment is simple. Additionally, the vehicle flow of this route is not too high, which ensured the safety of the experiments. The map of the determined test route is presented in Figure 4. The selected road is composed of a relatively gentle curve and a 4-km straight section. There are no obvious slopes in either the horizontal or vertical directions. The test section is completely closed, and there are no instances of turning vehicles around or crossing pedestrians along the whole section, excluding the starting and finishing positions at which vehicles can turn around at intersections controlled by traffic lights. The road is separated by a central separation belt, and the outside of the road is separated from the auxiliary road by a green belt. The speed limit on this road is 70 km/h, and the actual observed traffic flow is about 700 vehicles per hour. 

### 3.3. Cognitive Distraction Tasks

In this work, cognitive distraction tasks were divided into three types (as shown in Table 1), namely simple calculation (addition and subtraction), complex calculation, and the short-term memorization of a mobile phone number. These cognitive distraction subtasks not only conform to the actual state of cognitive distraction, but also have strong operability. Both simple and complex calculations refer to double-digit addition and subtraction. Simple calculations do not require borrowing, while complex calculations do. The test staff explained the calculation task to the participants and asked the driver to answer immediately. If the answer was correct, the next question would be started. If the answer was wrong, the test staff would repeat the question again. After the same question was asked for the second time, the next question was asked regardless of whether the answer was correct. Short-term memory refers to the driver remembering and repeating an 11-digit mobile phone number reported by the staff in a short amount of time. The participants had two chances to repeat the phone number before the next question was asked. During the test, the staff recorded the correctness rate of answers. To increase the coverage of the sample, different types of cognitive distraction subtasks were alternately presented.

### 3.4. Procedures

Before the test, the participants were required to have a trial run for approximately 20 min to be familiar with the experimental vehicle and the test environments. Then, the test staff introduced the cognitive distraction subtasks to the participants according to a pre-prepared plan. The participants performed the cognitive distraction subtasks according to the requirements while driving the test vehicle. Each subtask lasted about 25 s. The staff recorded the results of each subtask. After each subtask, the participants were free to manipulate the vehicle until the beginning of the next subtask. To alleviate driving fatigue, the participants could rest for 10 min after every 20 min of testing. During the test, the driver was required to strictly abide by the traffic rules and make safe driving as a priority at all times. In case of emergency, such as abnormal operation of the vehicle or equipment or the unsatisfactory condition of the participants, the test would be stopped immediately and the test vehicle would be safely parked in the emergency parking zone.

### 3.5. Obtained Data

After the experiments, 1500 sets of cognitive distraction driving data (the length of each set sequence was about 25 s, from the beginning of the subtask to the end) and 1600 sets of (the length of each set sequence was 25 s) normal driving data were obtained. To test whether the participants had effectively executed the cognitive distraction subtasks, the correctness rates of different participants performing different cognitive distraction tasks were counted. Each driver’s correctness rate while performing the cognitive distraction subtasks was greater than 85%, which indicates that the participants had a high degree of devotion when performing cognitive distraction subtasks. In other words, the test matched the requirements of simulating the actual distracted driving situation. 

### 3.6. Ethics and Authorization Statement

The experimental procedure was authorized by the research committee of Chang’an University, and the informed consent was received from each participant. In addition, the research did not require any extra license and the study complied with all correlative regulations. 

## 4. Wavelet Packet Feature Analysis Results

### 4.1. Wavelet Packet Characteristic Analysis of the Steering Wheel Angle Signal

**Figure 5:**
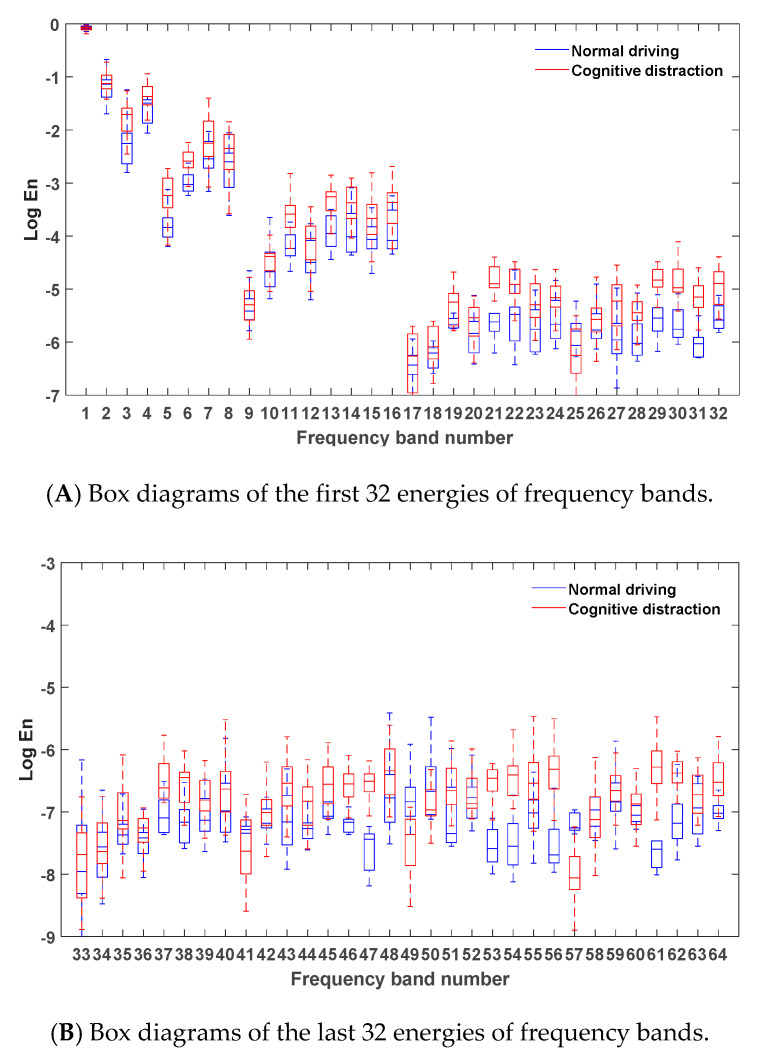
Box diagrams of the energies of frequency bands under different driving states.

After denoising the steering wheel angle signals in the normal driving data and the cognitive distraction driving data, six-layer wavelet packet analysis was performed to obtain the energies of 64 frequency bands. The energies of the frequency bands under both normal and cognitive distraction driving conditions were plotted by MATLAB software, and the results are exhibited in Figure 5.

Figure 5 reveals that the energy of the steering wheel angle signal was mostly concentrated in the first frequency band, which indicates that the steering wheel angle signal was mainly focused in the low-frequency region (0–0.2 Hz). Since the frequency and amplitude of the steering operation on a straight road section were relatively small in the time domain, the energy distribution in the low-frequency band was comparatively high. Due to the decrease in the driver’s ability to control the steering wheel while engaged in distracted driving, a relatively more frequent steering operation may have emerged. Hence, more than 95% of the energy of the frequency bands in the cognitive distraction driving state was greater than that in the normal driving state. 

Via comparative analysis, it was found that the energies of three frequency bands of 21, 47, and 61 in the cognitive distraction driving state were significantly higher than those in the normal driving state. The results of the one-way analysis of variance were p=0.00<0.05,F(1,3098)=4536.926, p=0.00<0.05,F(1,3098)=5032.314, and p=0.00<0.05,F(1,3098)=5981.280, respectively, which indicated that the energy values of the steering wheel angle under normal and distracted driving were significantly different in these three frequency bands. The real frequency bands corresponding to these three frequency bands were the 30th (4.53–4.69 Hz), 57th (8.75–8.91 Hz), and 35th (5.15–5.31 Hz) bands. Therefore, these three frequency bands were determined as the new characteristic parameters of the steering wheel angle signal. 

### 4.2. Wavelet Packet Characteristic Analysis of the Steering Wheel Angular Velocity Signal

**Figure 6:**
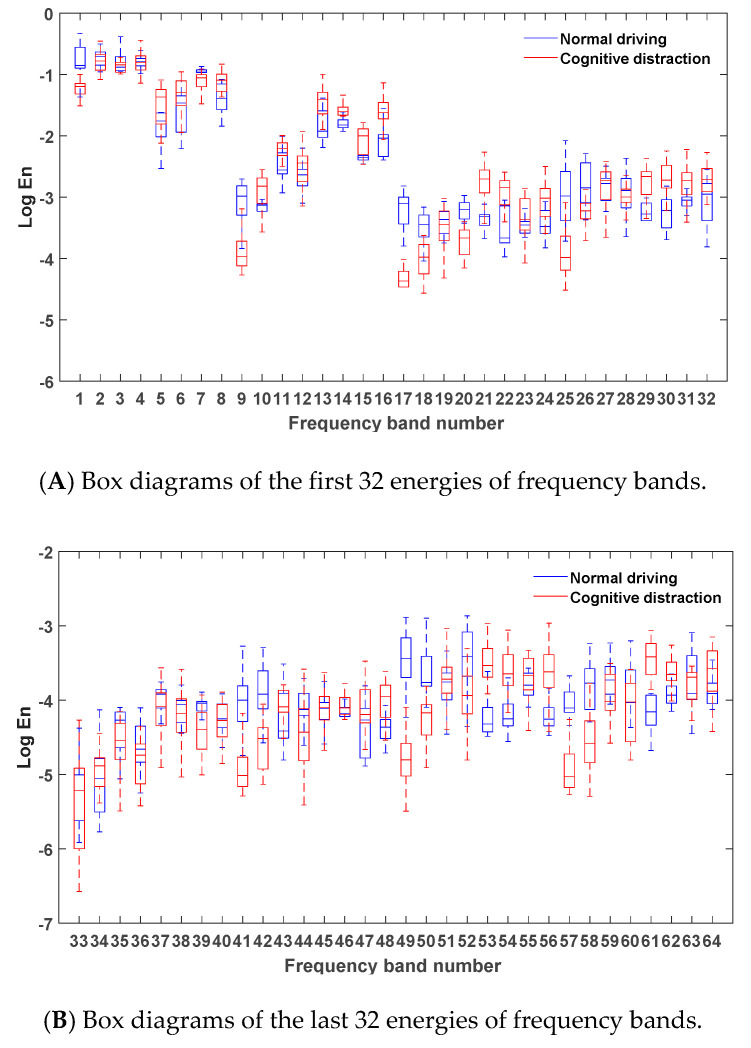
Box diagrams of the energies of frequency bands under different driving states.

After denoising the steering wheel angular velocity signals in the normal and cognitive distraction driving data, six-layer wavelet packet analysis was performed to obtain the energies of 64 frequency bands. The energies of the frequency bands under both normal and cognitive distraction driving conditions were plotted, and the results are presented in Figure 6.

Figure 6 reveals that the energy of the steering wheel angular velocity signal was mostly concentrated in the first four frequency bands, which indicates that the steering wheel angular velocity signal was primarily focused in the low-frequency region (0–0.5 Hz). Since the frequency and amplitude of the steering operation on a straight road section were relatively small in the time domain, the energy distribution in the low-frequency band was comparatively high. Due to the decrease in the driver’s ability to control the steering wheel while engaging in distracted driving, a relatively more frequent steering operation may have emerged. Hence, more than 80% of the energy of the frequency bands in the cognitive distraction driving state was greater than that in the normal driving state. 

Via comparative analysis, it was found that the energies of the four frequency bands of 1, 17, 49, and 57 in the normal driving state were significantly higher than those in the cognitive distraction driving state, whereas the energy of frequency band 61 in the cognitive distraction driving state was significantly higher than that in the normal driving state. The results of the one-way analysis of variance were p=0.00<0.05,F(1,3098)=3235.941, p=0.00<0.05,F(1,3098)=4125.374, p=0.00<0.05, F(1,3098)=6032.306, p=0.00<0.05, F(1,3098)=4451.283, and p=0.00<0.05,F(1,3098)=4035.761, respectively, which indicated that the energy values of the steering wheel angular velocity under normal and distracted driving were significantly different in these five frequency bands. The real frequency bands corresponding to these four frequency bands were the 1st (0–0.16 Hz), 24th (3.59–3.75 Hz), 40th (6.09–6.25 Hz), 36th (5.47–5.62 Hz), and 35th (5.15–5.31 Hz) bands. Therefore, these five frequency bands were determined as the new characteristic parameters of the steering wheel angular velocity signal.

### 4.3. Wavelet Packet Characteristic Analysis of the Vehicle Yaw Rate Signal

**Figure 7:**
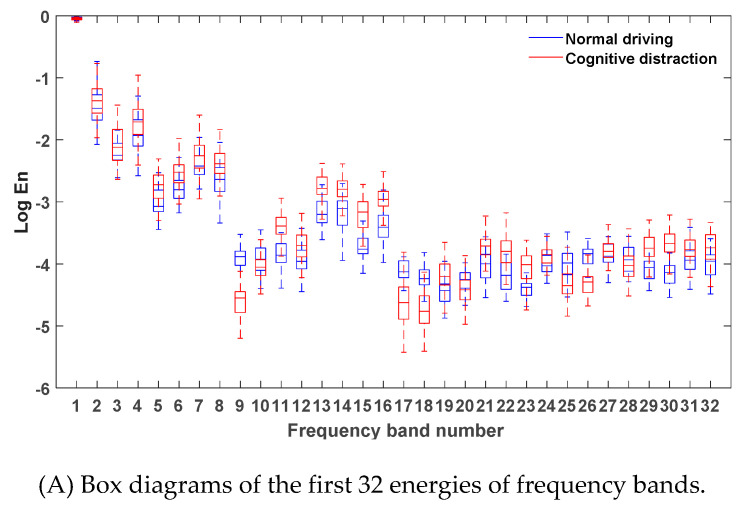

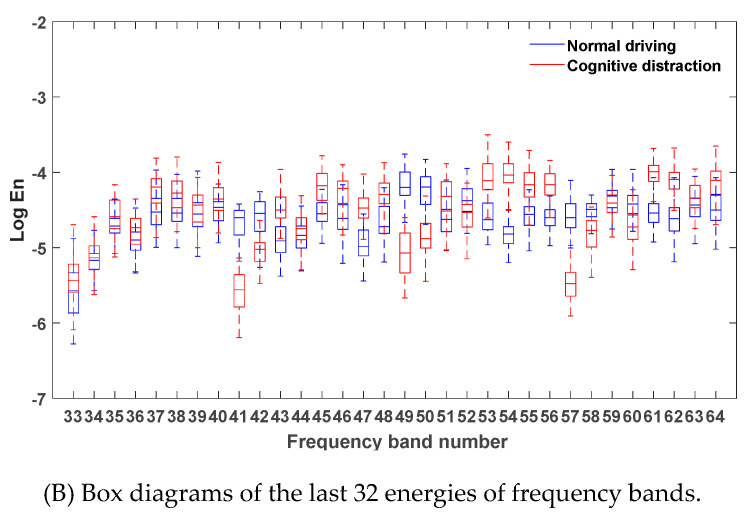
Box diagrams of the energies of the frequency bands under different driving states.

After denoising the vehicle yaw rate signals in the normal driving data and the cognitive distraction driving data, six-layer wavelet packet analysis was performed to obtain the energies of 64 frequency bands. The energies of the frequency bands under normal driving and cognitive distraction driving conditions were plotted, and the results are exhibited in Figure 7.

Figure 7 illustrates that the energy of the vehicle yaw rate signal was mostly concentrated in the first frequency band, which indicates that the steering wheel angular velocity signal was mainly focused in the low-frequency region (0–0.2 Hz). Since the frequency and amplitude of the steering operation on a straight road section were relatively small in the time domain, the energy distribution in the low-frequency band was comparatively high. Due to the decrease in the driver’s ability to control the steering wheel while engaging in distracted driving, a relatively more frequent steering operation may have emerged. Hence, more than 80% of the energy of the frequency band in the cognitive distraction driving state was greater than that in the normal driving state. 

Via comparative analysis, it was determined that the energies of four frequency bands of 9, 41, 49, and 57 in the normal driving state were significantly higher than those in the cognitive distraction driving state, while the energy of frequency band 54 in the cognitive distraction driving state was significantly higher than that in the normal driving state. The results of the one-way analysis of variance were p=0.00<0.05,F(1,3098)=3087.136, p=0.00<0.05, F(1,3098)=4081.719, p=0.00<0.05, F(1,3098)=4378.371, p=0.00<0.05,F(1,3098)=5408.614, and p=0.00<0.05, F(1,3098)=4819.320, respectively, which indicated that the energy values of the vehicle yaw rate under normal and distracted driving were significantly different in these five frequency bands. The real frequency bands corresponding to these five frequency bands were the 12th (1.71–1.87 Hz), 60th (9.22–9.38 Hz), 40th (6.09–6.25 Hz), 47th (7.18–7.34 Hz), and 36th (5.47–5.63 Hz) bands. Therefore, these five frequency bands were determined as the new characteristic parameters of the vehicle yaw rate signal.

### 4.4. Wavelet Packet Characteristic Analysis of the Vehicle Longitudinal Acceleration Signal

**Figure 8:**
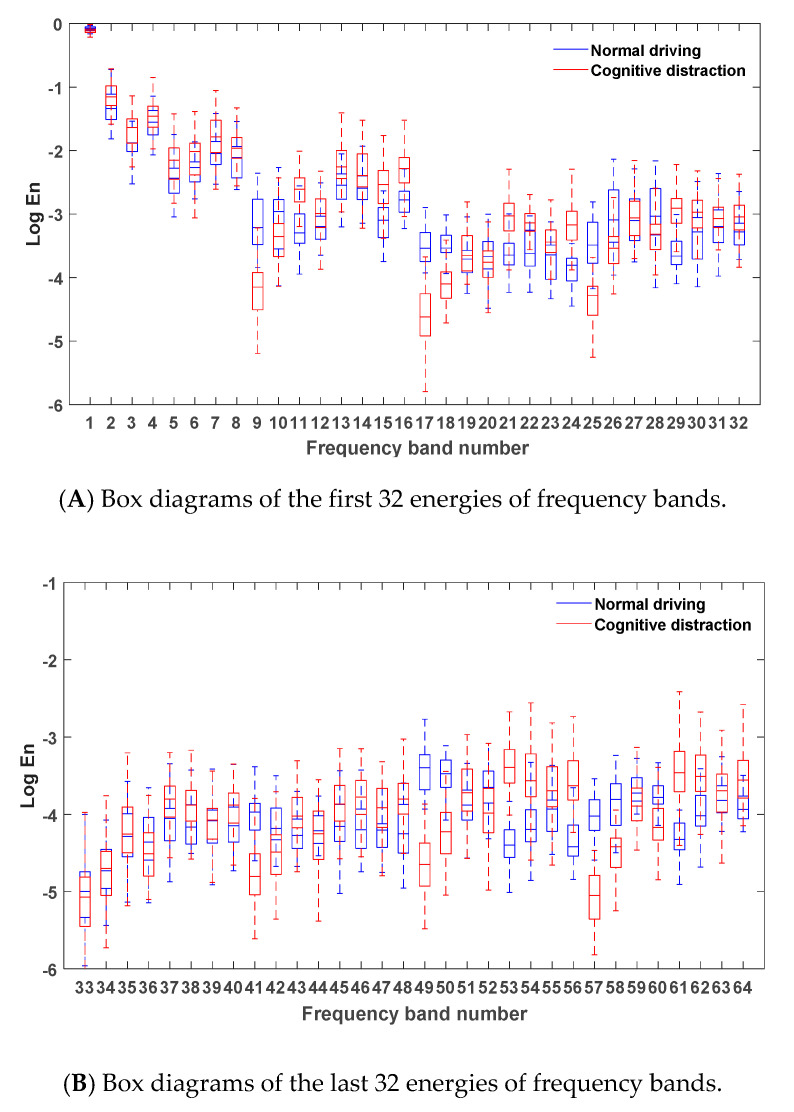
Box diagrams of the energies of the frequency bands under different driving states.

After denoising the vehicle longitudinal acceleration signals in the normal driving data and the cognitive distraction driving data, six-layer wavelet packet analysis was performed to determine the energies of 64 frequency bands. The energies of the frequency bands under both normal driving and cognitive distraction driving conditions were plotted, and the results are exhibited in Figure 8.

Figure 8 demonstrates that the energy of the vehicle yaw rate signal was mostly concentrated in the first frequency band, which indicates that the steering wheel angular velocity signal was mainly focused in the low-frequency region (0–0.2 Hz). Since the frequency and amplitude of throttle control on a straight road section were relatively small in the time domain, the energy distribution in the low-frequency band was comparatively high. Due to the decrease in the driver’s ability to control the throttle while engaging in distracted driving, a relatively more frequent throttle control may have emerged. Hence, more than 80% of the energy of the frequency band in the cognitive distraction driving state was greater than that in the normal driving state. 

Via comparative analysis, it was found that the energies of two frequency bands of 49 and 57 in the normal driving state were significantly higher than those in the cognitive distraction driving state, while the energy of frequency band 53 in the cognitive distraction driving state was significantly higher than that in the normal driving state. The results of the one-way analysis of variance were p=0.00<0.05, F(1,3098)=6283.648, p=0.00<0.05, F(1,3098)=5349.276, and p=0.00<0.05, F(1,3098)=5122.390, respectively, which indicated that the energy values of the vehicle longitudinal acceleration under normal and distracted driving were significantly different in these three frequency bands. The real frequency bands corresponding to these three frequency bands were the 40th (6.09-6.25 Hz), 46th (7.02-7.18 Hz), and 36th (5.47-5.63 Hz) bands. Therefore, these three frequency bands were determined as the new characteristic parameters of the vehicle longitudinal acceleration signal.

### 4.5. Wavelet Packet Characteristic Analysis of the Vehicle Lateral Acceleration Signal

**Figure 9:**
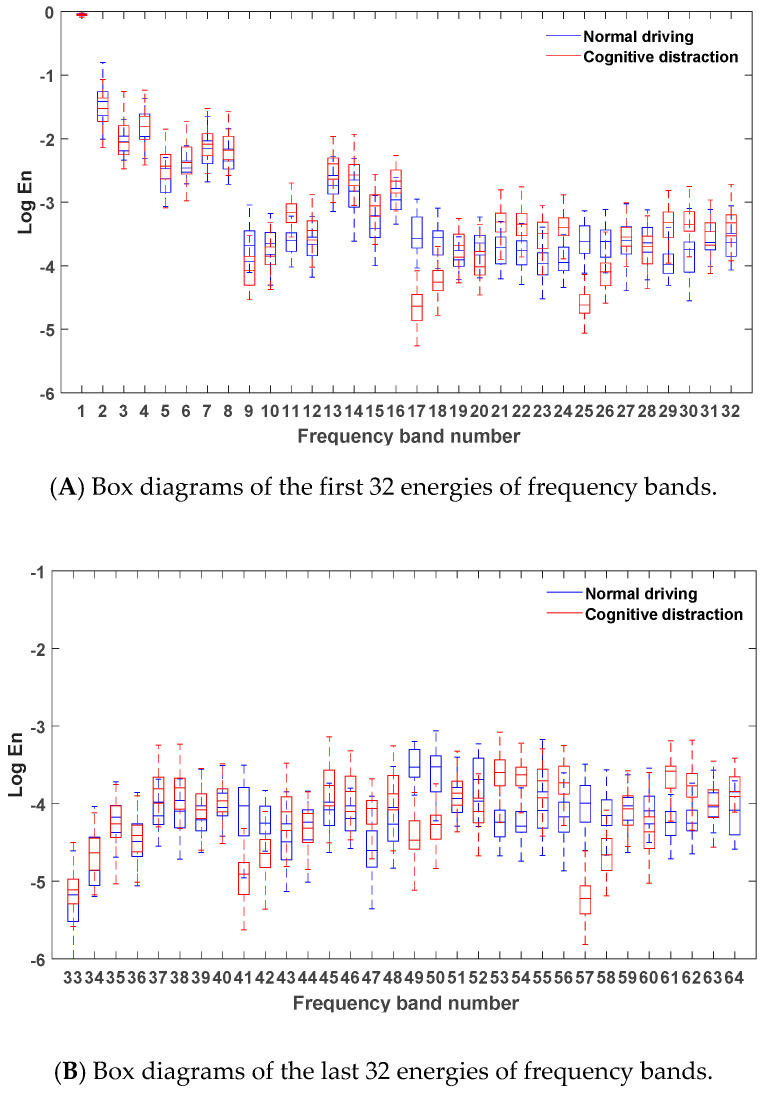
Box diagrams of the energies of frequency bands under different driving states.

After denoising the vehicle lateral acceleration signals in the normal driving data and the cognitive distraction driving data, six-layer wavelet packet analysis was performed to obtain the energies of 64 frequency bands. The energies of the frequency bands under normal driving and cognitive distraction driving conditions were plotted, and the results are shown in Figure 9.

Figure 9 illustrates that the energy of the vehicle yaw rate signal was mostly concentrated in the first frequency band, which indicates that the steering wheel angular velocity signal was mainly focused in the low-frequency region (0–0.2 Hz). Since the frequency and amplitude of throttle and steering control on a straight road section were relatively small in the time domain, the energy distribution in the low-frequency band was comparatively high. Due to the decrease in the driver’s ability to control the throttle and steering wheel while engaging in distracted driving, a relatively more frequent throttle and steering control may have emerged. Hence, more than 80% of the energy of the frequency bands in the cognitive distraction driving state was greater than that in the normal driving state. 

Via comparative analysis, it was found that the energies of the four frequency bands of 17, 25, 49, and 57 in the normal driving state were significantly higher than those in the cognitive distraction driving state, while the energy of frequency band 53 in the cognitive distraction driving state was significantly higher than that in the normal driving state. The results of the one-way analysis of variance were p=0.00<0.05, F(1,3098)=4292.553, p=0.00<0.05, F(1,3098)=4133.984, p=0.00<0.05, F(1,3098)=4588.643, p=0.00<0.05, F(1,3098)=6271.551, and p=0.00<0.05, F(1,3098)=3670.062, respectively, which indicated that the energy values of the vehicle lateral acceleration under normal and distracted driving were significantly different in these five frequency bands. The real frequency bands corresponding to these five frequency bands were the 24th (3.59–3.75 Hz), 20th (2.97–3.13 Hz), 40th (6.09–6.25 Hz), 46th (7.02–7.18 Hz), and 36th (5.47–5.63 Hz) bands. Therefore, these five frequency bands were determined as the new characteristic parameters of the vehicle lateral acceleration signal.

## 5. Cognitive Distraction Recognition Results

In this work, a total of 3100 effective samples including 1500 sets of cognitive distraction driving data and 1600 sets of normal driving data were collected. The total sample was distributed into a training set, a verification set, and a test set according to the ratio of 6:3:1: there were 1860 samples in the training set, 930 samples in the verification set, and 310 samples in the test set, as shown in Table 2. According to the wavelet packet analysis results presented in Section 4, 21 new characteristic parameters originating from the steering wheel angle, steering wheel angular velocity, vehicle yaw rate, vehicle longitudinal acceleration, and vehicle lateral acceleration were determined as critical features by which to distinguish normal driving and cognitive distraction driving. Therefore, the length of the training sample was 21.

### 5.1. Recognition Results with Different Time Windows

The earlier the recognition of the cognitive distraction of a driver, the more promptly the IDS will make the necessary intervention on vehicle control. However, cognitive distraction is a consecutive process. The accuracy of the recognition model will be reduced if the time window is set to be too short; in contrast, the accuracy may increase as the time window lengthens, but the IDSs will be slower to recognize distracted driving. Therefore, the recognition accuracy and time lag must be comprehensively considered to determine a reasonable length of the time window.

**Figure 10:**
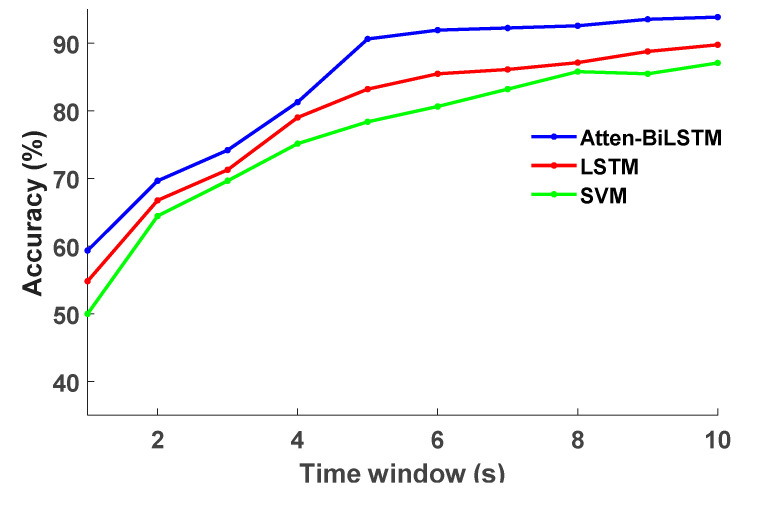
The recognition accuracy under different time window lengths.

In this study, different time window lengths were selected from 1 s to 10 s (the time interval was 1 s) to intercept the original data, and wavelet packet analysis was then employed to extract 21 new characteristic parameters to train the recognition model. The recognition accuracy of the training model under different time window lengths is presented in Figure 10, and the specific recognition results are reported in Table 3. Moreover, the recognition results of the SVM and LSTM models were compared with those of the proposed model.

According to the analysis presented in Figure 10 and Table 3, the Atten-BiLSTM model established in this study achieved the highest recognition accuracy at each time window length. With the increase in the time window length, the recognition accuracy of each model was gradually improved and dramatically increased between 1 and 5 s, after which the growth rate slowed. The recognition accuracy of the proposed model reached 90% when the time window length was 5 s, which could satisfy the needs of the IDS. As the time window length continued to increase, the promotion of recognition accuracy was not significant. Therefore, the optimal time window length was determined to be 5 s via the comprehensive consideration of the recognition accuracy and time lag. 

### 5.2. Recognition Model Performance Analysis

**Figure 11:**
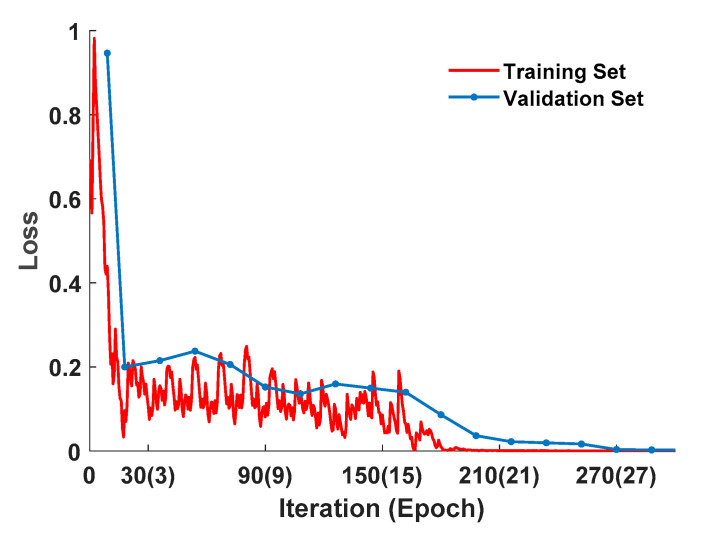
The loss value during model training.

According to the determined time window, the correlation performance of the established recognition model was analyzed including the accuracy rate, precision rate, recall rate, F1 scores, receiver operating characteristics (ROC) curve, and other indicators. Figure 11 presents the change of the loss value during the model training process with the number of training iterations under the time window length of 5 s.

It can be seen from Figure 11 that the loss value of the training set gradually decreased with the increase in the number of iterations during the training process, and it decreased rapidly within the first three epochs. As the number of training iterations continued to increase from 3 to 15 epochs, the loss value fluctuated. When the number of iterations exceeded 18 epochs, the loss value of the training set gradually stabilized. Similarly, the loss value of the verification set gradually decreased with the increase in the number of iterations, and the decreasing rate was faster within the first three epochs. As the number of iterations continued to increase, the decrease in the loss value of the verification set tended to be gentle, and when the number of iterations reached 27 epochs, the loss value dropped to a local minimum and stabilized at around 0.002. Therefore, the model training can be stopped after iteration for 27 epochs to prevent overfitting of the training model. 

The accuracy rate, precision rate, recall rate, and F1 scores of different recognition models were calculated, and the results are reported in Table 4. 

**Figure 12:**
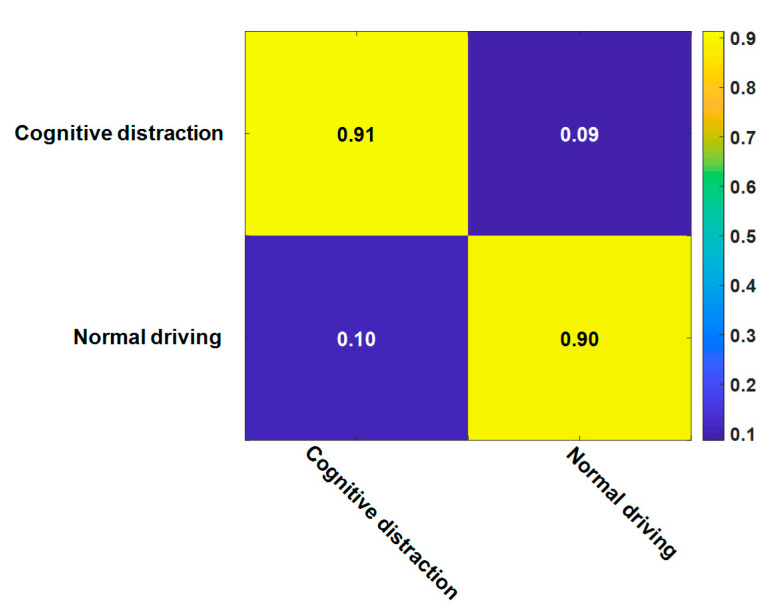
The confusion matrix for the attention (Atten)-BiLSTM model.

**Figure 13:**
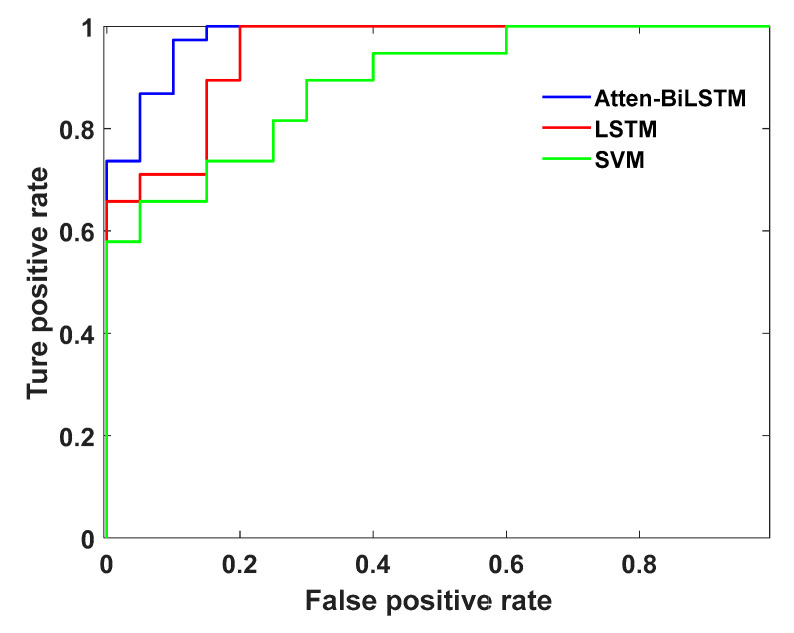
The receiver operating characteristics (ROC) curve for the Atten-BiLSTM model.

The results exhibited in Table 4 indicate that the performance of the Atten-BiLSTM recognition model was better than those of the LSTM and SVM models. The identification results of normal driving and cognitive distraction driving were specifically analyzed, and the confusion matrix is shown in Figure 12. It can be seen that the identification accuracy of cognitive distraction driving was higher than that of normal driving. The ROC curve based on the recognition results of different models is presented in Figure 13. 

The results displayed in Figure 13 demonstrate that the area under the curve (AUC) value of the Atten-BiLSTM recognition model was the largest of all models investigated; thus, the greater the AUC value, the better the capability of the recognition algorithm.

## 6. Discussions and Conclusions

In this study, an attention model was combined with a Bi-LSTM model to establish a recognition model of cognitive distraction driving. An on-road experiment was implemented, and data on the steering wheel angle, steering wheel angular velocity, vehicle yaw rate, vehicle longitudinal acceleration, and vehicle lateral acceleration of a vehicle under normal driving and cognitive distraction driving conditions were collected. To determine the time–frequency characteristics of the operation and vehicle status data, wavelet packet analysis was employed to analyze the collected data. Via comparative analysis, 21 characteristic frequency bands that can be used to distinguish between normal driving and cognitive distraction driving were ultimately extracted and determined. By using the 21 features as the input, the Atten-BiLSTM recognition model was trained and compared with the traditional SVM and LSTM models. The comparison results demonstrate that although all three models achieved high recognition accuracy, the proposed Atten-BiLSTM model provided more advantages for cognitive distraction driving recognition. The accuracy reached 90.64%, which was 7.42% higher than that of the LSTM algorithm and 12.26% higher than that of the SVM algorithm under the time window of 5 s. Other aspects of the recognition performance analysis results demonstrated that the proposed model can effectively distinguish between normal driving and cognitive distraction driving.

At present, driving simulators were generally employed to conduct cognitive distraction experiments. The application of the driving simulator for distracted driving research possessed many advantages over the real vehicle experiments, which included the safety, the experimental control, and the ease of data collection [39]. However, there were some possible disadvantages, including motion sickness, the scene authenticity, and most importantly, the validity. A large number of research had verified the absolute validity and relative validity of driving simulator results based on different research points, such as the verification of the driver’s longitudinal and lateral control performance under a distracted state [40]. Engstrom et al. [41] proved that the steering operation in the real vehicle test was more frequent than that in the simulator when the driver was under a distracted state. Reymond et al. [42] demonstrated that the maximum value of lateral acceleration in the real vehicle test was higher than that in the simulator. Considering that the driver’s cognitive load, operation data, and vehicle status data obtained in the actual vehicle test were different from in the driving simulator test under a distracted driving state, an on-road experiment was therefore implemented for the sake of collecting more realistic data, and the data were gathered from a test vehicle equipped with a steering wheel angle sensor, a gyro sensor, and a VBOX, which can provide support for the practical application of the recognition model of cognitive distraction driving.

In addition, an overwhelming majority of cognitive distraction recognition models were established based on the analysis of time domain features [43]. However, the driver’s operation data and vehicle movement data can be regarded as non-stationary signals, and it was necessary to catch the trend of the variation of the signal spectrum with time when dealing with non-stationary signals [44]. Therefore, in this study, 64 frequency bands were obtained via six-layer wavelet packet analysis, and the results indicate that the parameter signals were primarily concentrated in the low-frequency region. More than 80% of the energy of the frequency band in the cognitive distraction driving state was greater than that in the normal driving state. Via comparative analysis, 21 characteristic frequency bands that can be used to distinguish between normal driving and cognitive distraction driving were ultimately extracted and determined. These frequency bands include the 21st, 47th, and 61st bands of the steering wheel angle, the 1st, 17th, 49th, 57th, and 61st bands of the steering wheel angular velocity, the 9th, 41st, 49th, 54th, and 57th bands of the vehicle yaw rate, the 49th, 53rd, and 57th frequency bands of the vehicle longitudinal acceleration, and the 17th, 25th, 49th, 53rd, and 57th frequency bands of the vehicle lateral acceleration. The extracted features based on time–frequency characteristic analysis can provide support for improving the accuracy of the recognition model. 

The determination of the time window was a pivotal factor in the development of the recognition model of cognitive distraction. Sun et al. [45] proposed a driver’s distraction recognition model based on the LSTM algorithm and the time window was determined as 10 s. Zhou et al. [46] constituted a cognitive distraction identification model based on the random forest algorithm by using drivers’ eye-movement data and the time window was determined as 5 s. The time window of the distraction recognition model based on video data was usually short [47]. In summary, the different algorithms and different input data would influence the time window length setting of the recognition model. In practical application, the earlier the recognition of the cognitive distraction of a driver, the more promptly the IDS will make the necessary intervention on vehicle control. However, cognitive distraction was a continuous process. If the time window was set to be too short, the accuracy of the recognition model will be reduced; in contrast, the accuracy may increase as the time window lengthens, but the IDSs will be slower to recognize distracted driving. Therefore, the recognition accuracy and time lag must be comprehensively considered to determine a reasonable length of the time window. In this study, different time window lengths were selected from 1 to 10 s to intercept the original data, and wavelet packet analysis was then employed to extract 21 new characteristic parameters to train the recognition model. On the basis of the accuracy analysis of the recognition model under different time window lengths and while comprehensively considering the accuracy and time lag, the optimal time window was determined to be 5 s.

A few deficiencies in this work need to be ameliorated in future work. There was a difference between the actual state of cognitive distraction and the distraction state triggered by the designed secondary tasks. A future study will pay close attention to the difference and collect drivers’ cognitive distraction under naturalistic driving. In addition, the recognition model parameters will be calibrated according to more sufficient data. 

## Figures and Tables

**Table 1:** Cognitive distraction tasks.

Types	Examples
Simple calculation	15+4; 89−6; 45+3;…
Complex calculation	64−38; 56+29; 32−88;…
Short-term memorization of a mobile phone number	Phone number: 18594154134;…

**Table 2:** Number of samples in each set.

Label	Training Sample	Verification Sample	Test Sample
Normal Driving	960	480	160
Cognitive Distraction	900	450	150

**Table 3:** The recognition accuracy under different time window lengths.

Recognition Results (%)	1.0 s	2.0 s	3.0 s	4.0 s	5.0 s	6.0 s	7.0 s	8.0 s	9.0 s	10.0 s
Atten-BiLSTM	59.35	69.67	74.19	81.29	90.64	91.93	92.25	92.58	93.54	93.87
LSTM	54.83	66.77	71.29	79.03	83.22	85.48	86.13	87.14	88.78	89.78
SVM	50.00	64.45	69.67	75.16	78.38	80.64	83.22	85.80	85.48	87.09

**Table 4:** The recognition performance of different models.

Model	Accuracy Rate	Precision Rate	Recall Rate	F1 Scores
Atten-BiLSTM	90.64%	91.33%	89.5%	90.42%
LSTM	83.22%	84%	81.82%	82.90%
SVM	78.38%	80%	76.43%	78.17%
